# Factors Associated with Knowledge and Awareness of Stroke Among the Jordanian Population: A Cross-Sectional Study

**DOI:** 10.12688/f1000research.74492.1

**Published:** 2021-12-06

**Authors:** Muna Barakat, Husam A. AlSalamat, Feras Jirjees, Hala Al-Obaidi, Zainab k. Hussain, Seif El Hadidi, Sara Mansour, Diana Malaeb, Hassan Hosseini

**Affiliations:** 1 Department of Clinical Pharmacy and therapeutics, Faculty of Pharmacy, Applied Science Private University, Amman, 11931, Jordan; 2 Department of Basic Medical Sciences, Faculty of Medicine, Al-Balqa Applied University, Al-Salt, 19117, Jordan; 3 Department of Biopharmaceutics and Clinical Pharmacy, School of Pharmacy, University of Jordan, Amman, 11942, Jordan; 4 College of Pharmacy, University of Sharjah, Sharjah, 27272, United Arab Emirates; 5 College of pharmacy, Ajman University, Ajman, United Arab Emirates; 6 Department of Biology, College of Science, University of Baghdad, Baghdad, Iraq; 7 Faculty of Pharmaceutical Sciences and Pharmaceutical Industries, Future University in Egypt, Cairo, Egypt; 8 School of Pharmacy, Lebanese International University, Beirut, Lebanon; 9 Life Sciences and Health Department, Paris-Est University, Paris, France

**Keywords:** Awareness, Factors, Knowledge, Jordan, Stroke

## Abstract

**Background and objective**: Stroke is the second leading cause of death in Jordan and over the world. Knowledge and awareness towards stroke play a crucial role in the management and prevention of its complications. This study aims to assess the knowledge and awareness about stroke among the Jordanian population and determine factors associated with stroke awareness.

**Methods:** This cross-sectional study through a web-based anonymous questionnaire that needed 10 minutes to be completed. It examined sociodemographic characteristics and recognition of the risk factors, warning signs, stroke consequences, and early response to stroke symptoms. Logistic regression analysis identified the factors associated with poor knowledge of stroke.

**Results:** A total of 573 Jordanian adults participated in this study. The participant's ability to identify at least one early symptom of stroke and the proper response to the symptoms were significantly correlated with the educational level (OR of 3.4 and 2.5, respectively). At least one consequence of stroke was significantly associated with different demographic factors such as gender, socioeconomic income, females versus males and those with medium income versus low income had significantly higher odds (OR of 6.6 and 4.1, respectively).

**Conclusion:** This study revealed a good knowledge and awareness level about stroke among Jordanians correlated mainly with their educational level. Therefore, new strategies should be considered to decrease the prevalence of stroke in Jordan, including the need for engagement in enhanced awareness campaigns.

## Introduction

Stroke is the second cause of death worldwide, with approximately 11% of total deaths and is the leading cause of serious and permanent disability ( WHO, 2020, Katan, 2018). Moreover, in the past decades, the prevalence of stroke has increased more in developing countries than in developed countries due to several recent changes in the segment of countries (Feigin VL, 2009, Roth GA, 2020). For instance, stroke represents a major cause of disability and death in the last three decades in Jordan as a large Middle Eastern country. This surge has been linked to the prevalence of behavioral risk factors such as smoking, insufficient physical activity, and an unhealthy diet ( Vos et al., 2020, Ministry of Health, 2020).

Primary prevention of cerebrovascular accidents is essential to minimize stroke occurrence. It is achieved through different means, focusing on identifying associated risk factors, initiating prophylactic measures, and increasing patient awareness. Educational programs directed towards the community are among the best preventive measures; thus, an accurate assessment of comprehensive knowledge of stroke and its associated trigger factors is needed ( Sug Yoon et al., 2001, Trobbiani et al., 2013, Hatzitolios et al., 2014, Morren and Salgado, 2013, Pandian et al., 2005). In addition to improving the patients’ quality of life, knowledge will prevent healthcare professionals from being overwhelmed when stroke cases present to the emergency room at an early stage ( Awad and Al-Nafisi, 2014), noting that 80% of stroke cases are preventable if necessary precautions and actions are taken ( Vincent-Onabajo et al., 2015).

Globally, there is a lack of knowledge about stroke modifiable risk factors as unhealthy behaviors, obesity, smoking, and uncontrolled chronic diseases ( Medeiros et al., 2012, Boehme et al., 2017, Farrag et al., 2018). The accurate identification of stroke early symptoms is critical for quick and efficient medical interventions and the reduction of neuro-deficit complications as well as mortality ( Müller-Nordhorn et al., 2006, Stroebele et al., 2011a). Hence, in Low-Middle Income and developing countries, there is always a question about the public's understanding of stroke’s risk factors and related issues in terms of the condition’s risk, morbidity and mortality ( Stroebele et al., 2011c, Romero et al., 2008). Therefore, it is important to screen public characteristics and traits regarding lifestyle, behavior ( O'Donnell et al., 2016), educational level, smoking habits ( Hosseininezhad et al., 2017), and socioeconomic status ( Hawkes et al., 2015, Hosseininezhad et al., 2017).

Since stroke risk factors (i.e., history of hypertension or/and diabetes) are identifiable in individuals with low socioeconomic status, past medical history is also essential to investigate. Educational level, personal history of smoking, and high-income status have been associated with increased stroke knowledge ( Ramírez-Moreno et al., 2016). Gender is another factor to consider, as findings are contradictory. Indeed, several studies reported that women are more likely to present non-traditional stroke warning signs, develop stroke, and go late to the emergency department compared to males ( Lisabeth et al., 2009, Mandelzweig et al., 2006, Roger et al., 2012); oppositely, others showed that women recognize all the five traditional warning signs and quickly call the emergency department ( Focht et al., 2014).

Although the assessment of knowledge study deems simple, the outcomes of such research segment positively impact the design and implementation of highly effective interventions based on accurate population-based data. Yet, no nationwide study has been conducted in Jordan to assess the public awareness towards stroke. This study aims to highlight public’ gaps in knowledge and to reveal practice-related misconceptions in Jordan as a Middle-Eastern Developing country.

## Methods

### Study design

This descriptive cross-sectional study was carried out on the Jordanian population across all regions, using an anonymous online survey. A snowball sampling method was applied to abide by the lockdown restrictions enforced by the Jordanian Government (2020). An electronic questionnaire was created on Google forms and distributed to the Jordanian internet users (n= 6.5 million) via digital platforms (i.e., WhatsApp, LinkedIn, and Facebook) and made available online from February 2021 to April 2021. Participation in this study was voluntary and anonymous. Participants above 18 years of age were eligible; those with a history of stroke were excluded. The anonymity of the participants was guaranteed during the data collection process. A written participant consent statement “Your participation in completing this questionnaire is highly appreciated” was given to the participants at the beginning of the survey. If the participants were willing to proceed with the survey, they approved their consent. If not, they selected “disagree to participate” and did not continue with the survey questions. Potential participants who completed the survey were considered to have given informed consent for their participation in the study. Ethics approval for the study was obtained from the Faculty of Pharmacy, Applied Science Private University, Amman, Jordan (Approval Number: 2021-PHA-9).

### Sample size calculation

Based on another study, which concluded that around 71.8% of the participants were able to identify at least 3 out of 5 stroke risk factors ( Sadighi et al., 2018a), and in the absence of similar studies in Jordan, the Epi Info software version 7.2 (population survey) calculated a minimum sample of 312 participants at a confidence level of 95%. The reason for oversampling is to take into account patients’ refusal.

### Questionnaire

The questionnaire was in Arabic, the native language of Jordan and designed in a plain Arabic language. The expected filling time of the questionnaire is 20 minutes. This survey was developed based on previous literature ( Sadighi et al., 2018b, Han et al., 2019b). Participants filled it out without the help of investigators to avoid any potential influence when answering the questions.

The first section of the questionnaire covered the sociodemographic and socioeconomic factors, including age, smoking status, marital status (married versus others), employment status (employed versus not employed), family income, residence (urban versus rural), educational level, past medical history (e.g., hypertension, diabetes mellitus, dyslipidemia). Age was categorized into four groups (18-29, 30-49, 50-70, and above 70 years). The family income per month was divided into three financial categories: low (<400 JOD), intermediate (400-1000 JOD), and high (>1000 JOD), as 1 JOD equals 1.4 US Dollars ( Ahmed et al., 2019).

The second section assessed the general knowledge about stroke. Respondents answered the following statements: stroke 1) affects the brain, 2) is common among the elderly, 3) is contagious, 4) is hereditary, and 5) and can be prevented. This section also evaluated awareness about stroke risk factors, including hypertension, smoking, alcohol consumption, dyslipidemia, diabetes, physical inactivity, heart disorders, obesity, old age, and psychosocial stress. Moreover, it examined knowledge of early warning signs: 1) sudden numbness or weakness of the face, arms, or legs, especially on one side of the body; 2) sudden confusion or difficulty speaking or understanding speech; 3) sudden visual impairment in one or both eyes; 4) sudden difficulty walking, dizziness, or loss of balance or coordination; and 5) sudden severe headache with no known cause. Participants were awarded one point per correct answer to the above statements ( Han et al., 2019a).

### Statistical analysis

Statistical analysis was performed using the Statistical Package for Social Sciences version (SPSS) 25.0. All continuous variables were presented as mean and standard deviation (SD), and categorical variables were presented as frequencies (n) and percentages (%). Binary logistic regression was performed to determine the factors associated with the ability to spontaneously answer at least one or more stroke risk factors, one or more warning signs, one or more consequences, and seeking an emergency room as soon as stroke develops. Variables with a p<0.2 in the bivariate analysis were included in the regression analysis. Results were presented as odds ratios (OR) and 95% CI. Statistical tests were two-tailed and reported statistically significant at p < 0.05.

## Results

### Sociodemographic characteristics of the participants

A total of 573 participants completed the questionnaire. Of which, 65.1% are females and 59.2% are married, Table 1. A total of 93.4% of participants had finished their third-level education, and 85.9% were living in urban areas. Regarding the medical history, the most reported concomitant diseases were dyslipidemia (21%), obesity (18%) and Hypertension (15.7%). 94.8% of the participants reported their familiarity with the term stroke, while 31.4% just knew the term when a family member had it.

**Table 1.** Participants’ sociodemographic characteristics, past medical history and familiarity with stroke.

Variables (N = 573)		Frequency (%)
**Sociodemographic characteristics**		
**Gender**	Male	200 (34.9)
	Female	373 (65.1)
**Age (years)**	<30	183 (31.9)
	30-49	270 (47.1)
	>50	120 (21)
**Residence area**	Urban	492 (85.9)
	Rural	81 (14.1)
**Marital status**	Single	206 (36)
	Married	339 (59.2)
	Divorced	20 (3.5)
	Widowed	8 (1.3)
**Educational level**	Scholar level	38 (6.6)
	University level	536 (93.4)
**Employment status**	Unemployed	227 (39.6)
	Employed	346 (60.4)
**Income level**	Low	149 (26)
	Medium	302 (52.7)
	High	122 (21.3)
**Smoking (≥1 year)**	Yes	190 (33.2)
**Past medical history**	Hypertension	92 (15.7)
	Diabetes Mellitus	47 (8)
	Dyslipidemia	123 (21)
	Arrhythmia	86 (14.7)
	Kidney disease	27 (4.6)
	Peptic ulcer	59 (10)
	Depression	46 (8)
	Obesity	105 (18)
**Familiarity with stroke**	Ever heard of stroke	543 (94.8)
	History of stroke in the family	180 (31.4)
	Personally know someone with stroke	441 (77)

### Stroke knowledge

**Figure 1.**
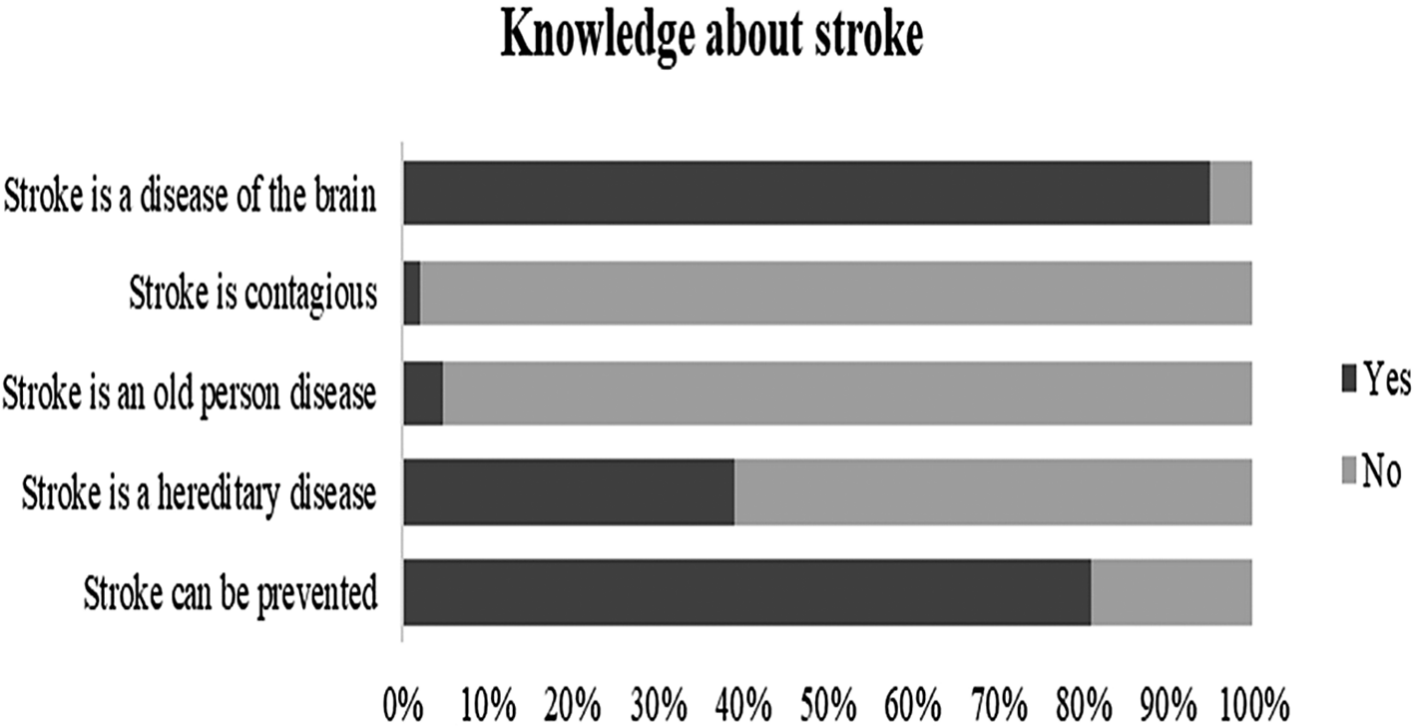
Assessment of stroke knowledge.

**Figure 2.**
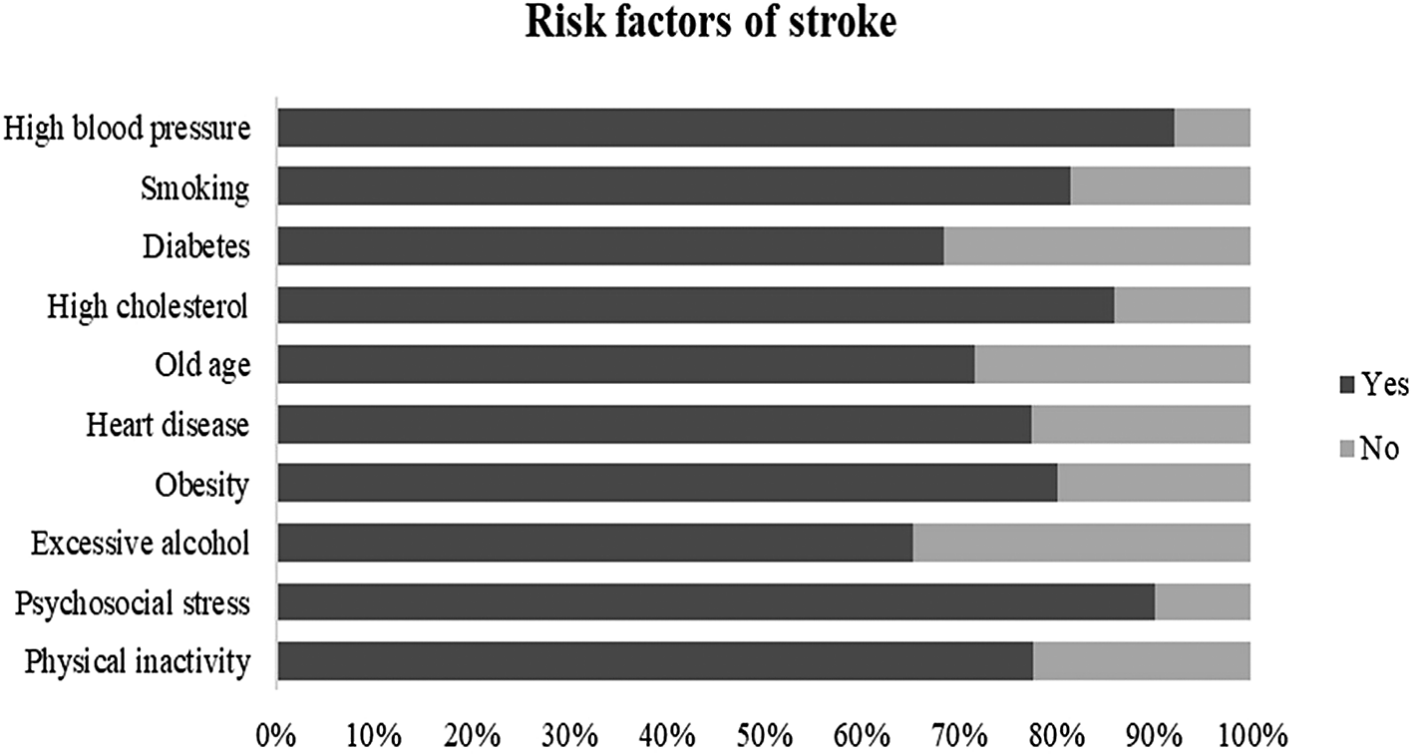
Identification of stroke risk factors.

**Figure 3.**
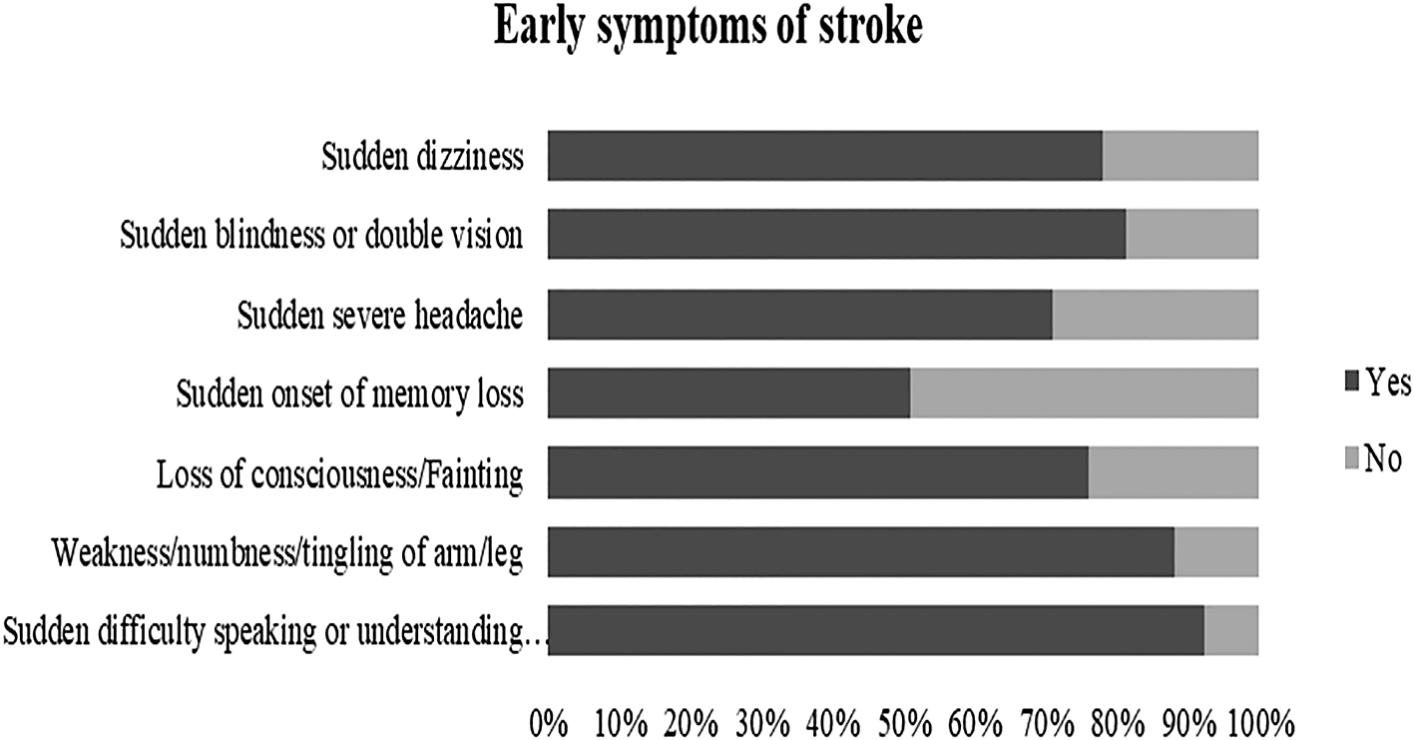
Awareness of stroke early symptoms.

The sample showed a satisfactory overall level of knowledge about stroke ( Figure 1 and Table 2). Nearly 95% of the participants mentioned that the brain is the primary organ of the body affected by stroke and 81% were aware of its possible prevention. In the question about risk factors, 92.1% believed that high blood pressure is the most common risk factor of stroke, followed by psychosocial stress (90.1%) and dyslipidemia (86%), Figure 2. The most identified warning signs were “Sudden difficulty in speaking or understanding speech” as 92.3% and “Sudden weakness/numbness/tingling” as 88%, Figure 3.

**Table 2.** Number of risk factors, early symptoms and consequences identified by the participants.

Variables (n = 573)		Frequency (%)	Cumulative, Frequency (%)
**Number of correct responses in the general knowledge**	Less than two	4 (0.7)	4 (0.7)
	Two	7 (1.2)	11 (1.9)
	Three	63 (11)	74 (12.9)
	Four	236 (41.2)	310 (54.1)
	Five	263 (45.9)	573 (100)
**Number of risk factors identified**	Zero	11 (1.9)	11 (1.9)
	One	1 (0.2)	12 (2.1)
	Two	14 (2.4)	26 (4.5)
	Three	17 (3)	43 (7.5)
	Four	23 (4)	66 (11.5)
	Five	28 (4.9)	94 (16.4)
	Six	48 (8.4)	142 (24.8)
	Seven	42 (7.3)	184 (32.1)
	Eight	79 (13.8)	263 (45.9)
	Nine	97 (16.9)	360 (62.8)
	Ten	213 (37.2)	573 (100)
**Number of early symptoms identified**	Zero	26 (4.5)	26 (4.5)
	One	7 (1.2)	33 (5.8)
	Two	24 (4.2)	57 (9.9)
	Three	23 (4)	80 (14)
	Four	52 (9.1)	132 (23)
	Five	106 (18.5)	238 (41.5)
	Six	129 (22.5)	367 (64)
	Seven	206 (36)	573 (100)
**Number of consequences identified**	Zero	11 (1.9)	11 (1.9)
	One	12 (2.1)	23 (4)
	Two	15 (2.6)	38 (6.6)
	Three	53 (9.2)	91 (15.9)
	Four	123 (21.5)	214 (37.3)
	Five	359 (62.7)	573 (100)

**Figure 4.**
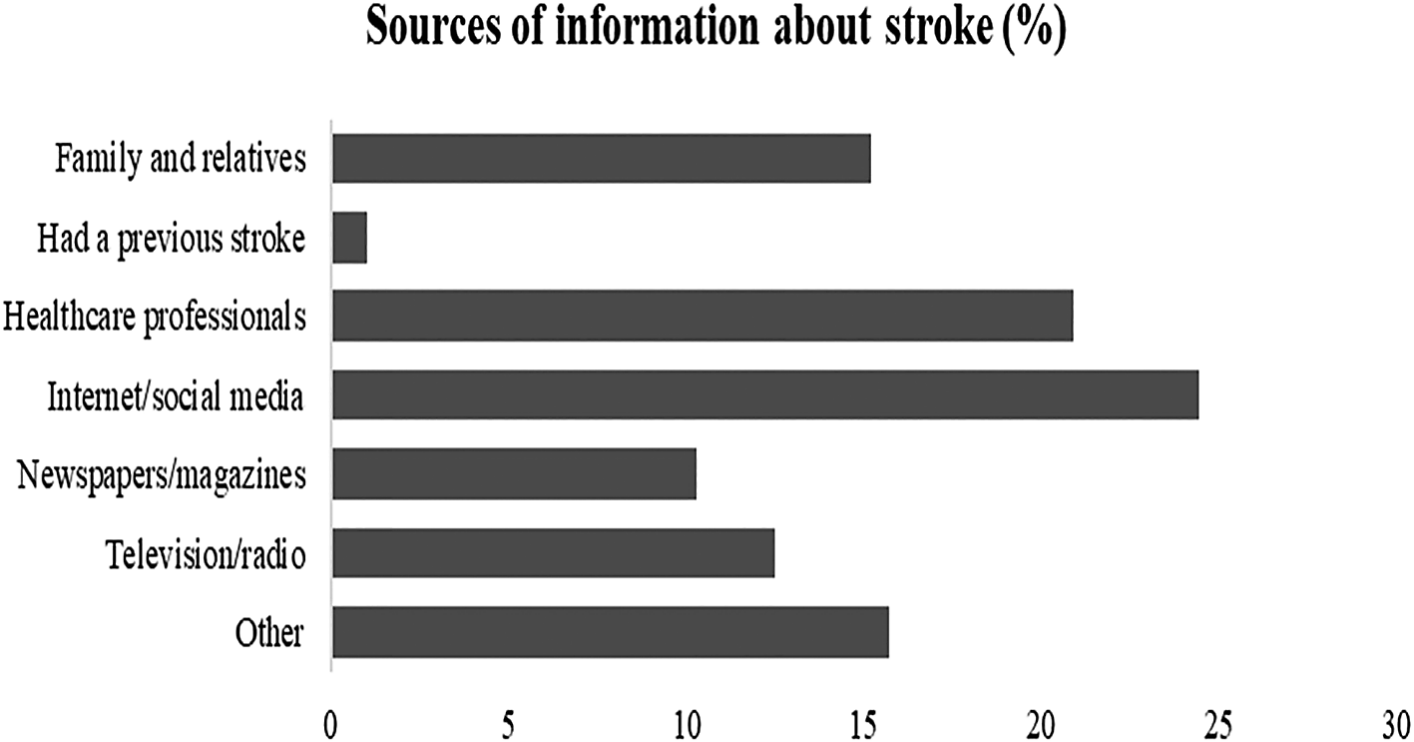
Evaluation of sources of information about stroke.

Internet/social media was the primary source of information about stroke as described by 24.4% of the respondents, followed by healthcare professionals as reported by 20.9% and family/relatives as 15.2%, Figure 4.

### Bivariate analysis

A total of 37.2 % identified all the risk factors appropriately, 36% recognized all the symptoms, and 62.7% stated all possible consequences of stroke. A significantly higher proportion of participants who are residents of the urban areas versus rural (86.5% vs. 13.5%) correctly identified the risk factors. Moreover, a significantly higher proportion of participants with university level of education compared to scholar level (94% vs. 6%) and those with no history of diabetes compared to having diabetes (92.3% vs. 7.7%) recognized at least one warning symptom of stroke. A significantly higher proportion of females versus males (65.8% vs. 34.2%) and those residing in urban areas vs. rural areas (86.7% vs. 13.3%) correctly identified the consequences emerging from stroke ( Table 3).

**Table 3.** Association of risk factors, early symptoms and consequences of stroke with the sociodemographic characteristics and past medical history.

Variables (n = 573)		Risk factor(s) identified (≥1)			Early symptom(s) identified (≥1)			Consequence(s) identified (≥1)		
		Yes (n = 562) n (%)	No (n = 11) n (%)	P-value	Yes (n = 547) n(%)	No (n = 26) n (%)	P-value	Yes (n = 562) n(%)	No (n = 11) n (%)	P-value
**Sociodemographic characteristics**										
Gender	Male	194 (34.5)	6 (54.5)	0.204	187 (34.2)	13 (50)	0.098	192 (34.2)	8 (72.7)	**0.020**
	Female	368 (65.5)	5 (45.5)		360 (65.8)	13 (50)		370 (65.8)	3 (27.3)	
Age (years)	<30	178 (31.7)	5 (45.5)	0.614	176 (32.2)	7 (27)	0.723	176 (31.3)	7 (63.6)	0.190
	30-49	265 (47.1)	5 (45.5)		255 (46.6)	15 (57.7)		267 (47.5)	3 (27.4)	
	50-70	114 (20.3)	1 (9)		111 (20.3)	4 (15.3)		114 (20.3)	1 (9.1)	
	>70	5 (0.9)	0 (0)		5 (0.9)	0 (0)		5 (0.9)	0 (0)	
Residence area	Urban	486 (86.5)	6 (54.5)	**0.012**	473 (86.5)	19	0.077	487 (86.7)	5 (45.5)	**0.002**
	Rural	76 (13.5)	5 (45.5)		74 (13.5)	7 (27)		75 (13.3)	6 (54.5)	
Marital status	Single	201 (35.8)	5 (45.5)	0.859	201 (36.7)	5 (19.2)	0.127	201 (35.8)	5 (45.5)	0.859
	Married	333 (59.2)	6 (54.5)		320 (58.5)	19 (73)		333 (59.2)	6 (54.5)	
	Divorced	20 (3.6)	0 (0)		19 (3.5)	1 (3.9)		20 (3.6)	0 (0)	
	Widowed	8 (1.4)	0 (0)		7 (1.3)	1 (3.9)		8 (1.4)	0 (0)	
Educational level	Scholar	36 (6.4)	2 (18.2)	0.161	33 (6)	5 (19.2)	**0.023**	36 (6.4)	2 (18.2)	0.161
	University	526 (93.6)	9 (81.8)		514 (94)	21 (80.8)		526 (93.6)	9 (81.8)	
Employment status	Unemployed	220 (39.1)	7 (63.6)	0.123	215 (39.3)	12 (46.2)	0.485	221 (39.3)	6 (54.5)	0.358
	Employed	342 (60.9)	4 (36.4)		332 (60.7)	14 (53.8)		341 (60.7)	5 (45.5)	
Income level	Low	145 (25.8)	4 (36.4)	0.211	139 (25.4)	10 (38.5)	0.237	143 (25.5)	6 (54.5)	0.123
	Medium	295 (52.5)	7 (63.6)		289 (52.8)	13 (50)		298 (53)	4 (36.4)	
	High	122 (21.7)	0 (0)		119 (21.8)	3 (11.5)		121 (21.5)	1 (9.1)	
Smoking (≥1 year)	No	374 (66.5)	9 (81.8)	0.355	366 (67)	17 (65.4)	0.872	376 (67)	7 (63.6)	0.759
	Yes	188 (33.5)	2 (18.2)		181 (33)	9 (34.6)		186 (33)	4 (36.4)	
**Past medical history**										
Hypertension	No	472 (84)	9 (81.8)	0.692	462 (84.5)	19 (73)	0.165	472 (84)	9 (81.8)	0.692
	Yes	90 (16)	2 (18.2)		85 (15.5)	7 (27)		90 (16)	2 (18.2)	
Diabetes Mellitus	No	516 (91.8)	10 (91)	1.000	505 (92.3)	21 (80.8)	**0.053**	516 (91.8)	10 (91)	1.000
	Yes	46 (8.2)	1 (9)		42 (7.7)	5 (19.2)		46 (8.2)	1 (9)	
Dyslipidemia	No	440 (78.3)	10 (91)	0.471	430 (78.6)	20 (77)	0.838	440 (78.3)	10 (91)	0.471
	Yes	122 (21.7)	1 (9)		117 (21.4)	6 (23)		122 (21.7)	1 (9)	
Arrhythmia	No	478 (85)	9 (81.8)	0.674	467 (85.4)	20 (77)	0.258	479 (85.2)	8 (72.7)	0.220
	Yes	84 (15)	2 (18.2)		80 (14.6)	6 (23)		83 (14.8)	3 (27.3)	
Kidney disease	No	536 (95.4)	10 (91)	0.415	522 (95.4)	24 (92.3)	0.350	536 (95.4)	10 (91)	0.415
	Yes	26 (4.6)	1 (9)		25 (4.6)	2 (7.7)		26 (4.6)	1 (9)	
Peptic ulcer	No	504 (89.7)	10 (91)	1.000	489 (89.4)	25 (96.1)	0.504	504 (89.7)	10 (91)	1.000
	Yes	58 (10.3)	1 (9)		58 (10.6)	1 (3.9)		58 (10.3)	1 (9)	
Depression	No	518 (92.2)	9 (81.8)	0.219	504 (92)	23 (88.5)	0.456	518 (92.2)	9 (81.8)	0.219
	Yes	44 (7.8)	2 (18.2)		43 (8)	3 (11.5)		44 (7.8)	2 (18.2)	
Obesity	No	458 (81.5)	10 (91)	0.698	444 (81)	24 (92.3)	0.198	458 (81.5)	10 (91)	0.698
	Yes	104 (18.5)	1 (9)		103 (19)	2 (7.7)		104 (18.5)	1 (9)	

Numbers in **bold** indicate significant p-values.

In terms of attitude, a significantly higher number of correct answers was associated with university compared to scholar level of education (94.3 % vs. 5.7%), who had a job versus unemployed (62.2% vs. 37.8%) and those with no history of diabetes compared to having diabetes (92.7% vs. 7.3%) ( Table 4).

**Table 4.** Association of response in case of facing somebody with acute symptoms of a stroke (identified by taking the patient to the hospital) and with sociodemographic characteristics, aand past medical history

Variables (n = 573)		Response in case of facing somebody with acute symptoms of stroke identified by taking the patient to the hospital		
		Yes (n = 510), n(%)	No (n = 63), n (%)	P-value
**Sociodemographic characteristics**				
Gender	Male	180 (35.3)	20 (31.7)	0.577
	Female	330 (64.7)	43 (68.3)	
Age (years)	<30	163 (32)	20 (31.7)	0.957
	30-49	241 (47.2)	29 (46)	
	50-70	101(19.8)	14 (22.3)	
	>70	5 (1)	0 (0)	
Residence area	Urban	440 (86.3)	52 (82.5)	0.422
	Rural	70 (13.7)	11 (17.5)	
Marital status	Single	186 (36.5)	20 (31.7)	0.472
	Married	300 (58.8)	39 (61.9)	
	Divorced	18 (3.5)	2 (3.2)	
	Widowed	6 (1.2)	2 (3.2)	
Educational level	School	29 (5.7)	9 (14.3)	**0.026**
	University	481 (94.3)	54 (85.7)	
Employment status	Unemployed	193 (37.8)	34 (54)	**0.014**
	Employed	317 (62.2)	29 (46)	
Income level	Low	128 (25.1)	21 (33.3)	0.213
	Medium	269 (52.7)	33 (52.4)	
	High	113 (22.2)	9 (14.3)	
History of smoking (≥1 year)	No	340 (66.7)	43 (68.3)	0.801
	Yes	170 (33.3)	20 (31.7)	
**Past medical history**				
Hypertension	No	430 (84.3)	51 (81)	0.493
	Yes	80 (15.7)	12 (19)	
Diabetes Mellitus	No	473 (92.7)	53 (84.1)	**0.019**
	Yes	37 (7.3)	10 (15.9)	
Dyslipidemia	No	402 (78.8)	48 (76.2)	0.631
	Yes	108 (21.2)	15 (23.8)	
Arrhythmia	No	437 (85.7)	50 (79.4)	0.185
	Yes	73 (14.3)	13 (20.6)	
Kidney disease	No	485 (95)	61 (96.8)	0.757
	Yes	25 (5)	2 (3.2)	
Peptic ulcer	No	455 (89.2)	59 (93.7)	0.274
	Yes	55 (10.8)	4 (6.3)	
Depression	No	470 (92.2)	57 (90.5)	0.643
	Yes	40 (7.8)	6 (9.5)	
Obesity	No	417 (81.8)	51 (81)	0.875
	Yes	93 (18.2)	12 (19)	

Numbers in **bold** indicate significant p-values.

### Multivariable analysis

When considering the identification of at least a risk factor as the dependent variable, the multivariable analysis showed that those residing in rural areas were less likely to identify a risk factor than those living in urban areas (OR = 0.2, p-value of 0.011).

The participant's ability to identify at least one early symptom of stroke as the dependent variable, university compared to the scholar level of education had significantly higher odds (OR = 3.4, p-value of 0.023), and diabetes was inversely associated with early symptoms identification (OR = 0.2, p-value of 0.008).

When considering the identification of at least one consequence of stroke as the dependent variable, females versus males and those with medium income versus low income had significantly higher odds (OR of 6.6 and 4.1 respectively). Moreover, residents of rural areas were less likely to identify stroke consequences compared to urban residents (OR = 0.1, p-value of 0.005).

**Figure 5.**
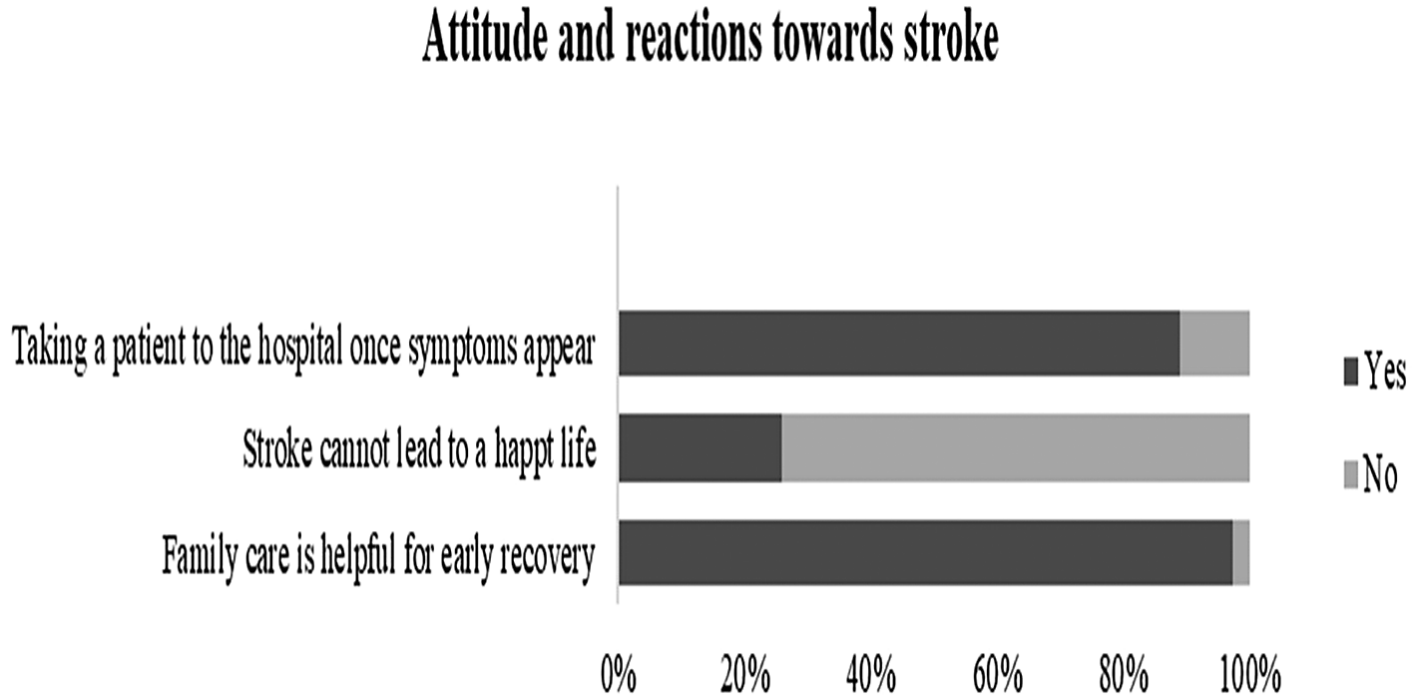
Knolwedge, attitude, and reactions of the participants towards stroke.

Concerning the response to stroke symptoms (by taking the patient to the hospital) as the dependent variable, university compared to scholar level of education and employed versus unemployed had significantly higher odds (OR of 2.5 and 1.8 respectively) whereas, having diabetes was associated with lower odds compared to no diabetes history (OR = 0.4) ( Table 5, Figure 5).

**Table 5.** Multivariate analysis.

Variables (n = 573)	β (SE)	OR (95% CI)	P-value
**Risk factor(s) identified (≥1)**			
Gender (female versus male*)	1.1 (0.6)	2.9 (0.8-10.2)	0.098
Residence area (rural versus urban*)	−1.5 (0.6)	0.2 (0.060-0.697)	**0.011**
Employment status (employed versus unemployed*)	1.2 (0.6)	3.4 (0.9-12.6)	0.062
**Early symptom(s) identified (≥1)**			
Educational level (university versus scholar*)	1.2 (0.5)	3.4 (1.1-9.8)	**0.023**
Residence area (rural versus urban*)	−0.8 (0.4)	0.4 (0.1-1.04)	0.063
Diabetes (yes versus no*)	−1.4 (0.5)	0.2 (0.07-0.68)	**0.008**
Obesity (yes versus no*)	1.3 (0.7)	3.7 (0.8-17.2)	0.093
**Consequence(s) identified (≥1)**			
Gender (female versus male*)	1.8 (0.7)	6.6 (1.6-26.9)	**0.008**
Residence area (rural versus urban*)	−1.8 (0.6)	0.1 (0.04-0.5)	**0.005**
Income level (medium versus low*)	1.4 (0.6)	4.1 (1.04-15.7)	**0.043**
Income level high versus low*)	1.7 (1.1)	5.5 (0.58-52.03)	0.137
**Taking a patient to a hospital**			
Educational level (university versus school*)	0.9 (0.4)	2.5 (1.1-5.5)	**0.030**
Employment status (employed versus unemployed*)	0.6 (0.2)	1.8 (1.1-3.1)	**0.028**
Diabetes (yes versus no*)	−0.9 (−0.3)	0.4 (0.18-0.85)	**0.018**

β, Beta; SE, standard error; OR; adjusted ratio; CI, confidence interval.

Logistic regression taking identification of stroke risk factors, stroke early symptoms, stroke consequences, response if faced with stroke as the dependent variables and sociodemographic factors (gender, residence area, educational level, employment status, and income level) as independent variables.

## Discussion

Public health literacy is a strong asset for a healthier community. As of August 2020, The U.S. Department of Health and Human Services (HHS) released Healthy People 2030, introducing an updated definition of personal health literacy as “the degree to which individuals can find, understand, and use information and services to inform health-related decisions and actions for themselves and others,” while organizational health literacy describes the degree to which organizations equitably enable individuals to pursue personal health literacy ( Services, 2020, Ancker et al., 2020). This study describes the levels of knowledge and awareness related to stroke among individuals from the general Jordanian population.

Most participants in our study expressed excellent knowledge regarding stroke, particularly being related to the brain, not contagious, not old-age specific, not hereditary, and being preventable. Additionally, most participants identified at least one risk factor, one consequence, and one symptom related to stroke. Compared to similar literature ( Sug Yoon et al., 2001, Pancioli et al., 1998, Croquelois and Bogousslavsky, 2006, Reeves et al., 2008), our outcome measures of stroke health literacy are higher, mainly that all knowledge, risk factors, symptoms, and consequences related to stroke were identified by more than 50% of the study sample.

In our study, 98.1% of participants identified at least one risk factor related to stroke. In comparison, previous studies have reported 85.4% among 390 participants in Lebanon ( Khalil and Lahoud, 2020), 76.2% among 822 participants in Australia ( Sug Yoon et al., 2001), and 59.6% among 2884 participants in Spain ( Segura et al., 2003). Conversely, other previous studies have demonstrated poor knowledge of stroke risk factors and symptoms in the general population ( Jones et al., 2010, Stroebele et al., 2011b, Nicol and Thrift, 2005). According to the latest 2021 update from the American Heart Association, risk factors related to stroke are high blood pressure, hyperglycemia, obesity, renal dysfunction, and hyperlipidemia, in addition to 47% being attributed to behavioral risk factors such as sedentary lifestyle, smoking, and an unhealthy diet ( Alonso et al., 2021). At the same time, 30% were attributed to air pollution worldwide ( Collaborators and Ärnlöv, 2020). Most identified risk factors related to stroke in our study were hypertension, psychological stress, hypercholesterolemia, smoking, and obesity, with percentages exceeding 80%. Unlike a previous 2014 Jordanian study of 1854 participants, which reported getting older (58.8%), previous stroke (56.6%), and hypertension (56.0%) as most commonly identified risk factors in their study ( Madae’en et al., 2013). This shows a more confident trend in identifying risk factors related to stroke among our study participants. Moreover, hypertension (48.2%), followed by stress (43.1%), were identified as risk factors among participants in a study from Lebanon (43.1%) ( Khalil and Lahoud, 2020). Similarly, among 469 participants in a study from Morocco, hypertension (55.7%), followed by stress (48.8%), were identified as risk factors for stroke ( Kharbach et al., 2020b). Despite being one of the most common modifiable risk factors for stroke, Diabetes Mellitus was relatively less identifiable by our study participants (68.4%). This finding has been reported elsewhere in previous studies ( Kharbach et al., 2020a).

Also, participants in our study expressed a higher percentage recalling at least one stroke symptom (95.5%) compared to studies in Portugal (74.2%) ( Duque et al., 2015), Norway (70.7%) ( Sundseth et al., 2014), Oman (68.0%) ( Al Shafaee et al., 2006), Korea (65%) ( Kim and Yoon, 1997), and Lebanon (68.2%) ( Khalil and Lahoud, 2020). Similarly, in a previous 2014 study from Jordan, (87.3%) of participants identified at least one sign and symptom related to stroke, which is still relatively high. ( Madae’en et al., 2013). Sudden difficulty speaking or understanding speech was the most frequently reported stroke symptom in our study (92.3%) compared to a previous study in Jordan (85.1%) ( Madae’en et al., 2013), and Australia (14.2%) ( Sug Yoon et al., 2001), and Ireland (54%) ( Hickey et al., 2009). However, sudden weakening of one side of the body was reported as relatively the most prevalent stroke symptom, as among Omani (65 %) ( Al Shafaee et al., 2006) and Nigerian (24.4%) populations ( Wahab et al., 2008).

Regarding their attitude toward stroke, participants in our study were encouraged to go to a hospital as soon as possible after a stroke is identified (89.0%), like a previous study that emphasized the need for immediate medical care for stroke patients ( Khalil and Lahoud, 2020). Among 400 participants in an earlier study from Oman, 73% of participants reported they would immediately go to the hospital emergency if they suspected a stroke ( Al Shafaee et al., 2006). However, percentages from international studies may vary, with only 47% claiming they would go to a hospital if they were suspicious of a stroke ( Jones et al., 2010). Adequate knowledge about risk factors, symptoms, and consequences related to stroke in our study could be attributed to the younger age and high level of education of the participants.

Our study findings showed that the female gender was attributed to better knowledge about stroke consequences than males, with no gender-specific difference in knowledge about risk factors and symptoms related to stroke. In a systematic review until 2008, the female gender was attributed to the better overall understanding of risk factors and symptoms related to stroke ( Stroebele et al., 2011b). Another study explained the male gender as a predictor of enhanced knowledge ( Wahab et al., 2008). Whether there are gender-specific variations in knowledge remains controversial and would need further in-depth causality assessments, as previous studies provide no consistent gender correlations in favor of such differences about stroke’s risk factors, symptoms, or consequences ( Park et al., 2006, Koçer et al., 2006, Pontes-Neto et al., 2008). Nevertheless, women tend to be more knowledgeable, express greater interest in health topics, and even spend more time seeking information than men do ( Horch and Wirz, 2005).

Moreover, our results revealed that living in an urban area was significantly associated with better awareness of stroke risk factors and consequences; this could be attributed to better access to information resources and health services than rural ones ( Joubert et al., 2008). In addition, participants who were well educated, employed, or diagnosed with diabetes in our study expressed willingness to promptly take a patient to hospital if they were suspicious of a stroke, which is somewhat expected, as better knowledge of consequences of a stroke would warrant prompt care. Furthermore, employment can warrant accessibility to seek medical help through insurance. While for diabetic patients, this might be attributed to their better knowledge of their disease status and consequences, as they often visit a healthcare provider for chronic medical care ( Bogoshi, 2003, Chukwuocha et al., 2018).

Concerning stroke information resources, no particular resource was regarded as major by the participants in our study, but rather relatively, the internet and social media (24.4%) were the most frequently used resource of information, followed by healthcare professionals (20.9%), and family or relatives (15.2%). This is rather alarming since publicly available health information across social media might not be evidence-based and often misinterpreted by the general public ( Suarez-Lledo and Alvarez-Galvez, 2021, Waszak et al., 2018).

### Limitations

Several limitations can be identified for this study. First, an online Google survey is subject to a security breach, yet password protection for editing privileges was implemented and accessible by the research team. Second, representation of the Jordanian population could be compromised, as the study tool warrants computer literacy, internet availability, an enhanced level of education to access and complete the online survey. Third, information bias related to the accessibility of resources on-demand can compromise response credibility. Fourth, selection bias related to the snowball collection technique might be an issue, with no random selection warranted. Residual confounding bias could arise from possible un-measured variables or responses to variables directly or indirectly related to stroke. Moreover, an online survey instead of a face-to-face meeting poses reliability and authenticity risks to the study data. The online survey included country-specific questions for Jordanians to complete, with a full description of the target population and inclusion criteria in the title and the invitation message. Considering the restriction measures during the COVID-19 pandemic, such a methodology was the best option.

## Conclusion

The general Jordanian population expresses good overall personal health literacy about risk factors, symptoms, and consequences related to stroke. Higher education levels, living in an urban residential area, and being employed were attributed to better knowledge about various aspects of the stroke. Through structured, reliable, evidence-based, and accessible health awareness resources, organizational health literacy is warranted to target individuals with inadequate personal health literacy related to stroke among the Jordanian population. Further nationwide studies could affirm more representative findings to the general Jordanian population.

## Data availability

Open Science Framework. Assessment of Knowledge, Awareness of Stroke, and the Factors Associated with Among Jordanian Population: A Cross-Sectional Study. DOI: https://doi.org/10.17605/OSF.IO/QZTV3.

This project contains the following data.

• Raw Data spss.sav
• STROBE_checklist_cross-sectional score.doc
• Stroke Awareness Questionnaire Final.docx

Data are available under the terms of the Creative Commons Attribution 4.0 International license (CC-BY 4.0).

## Authors' contributions

All authors were involved in all parts of the study and manuscript preparation, including literature search, study design, analysis of data, manuscript preparation, and review of the manuscript.
