# Supplementary material for: Factors Associated with Knowledge and Awareness of Stroke Among the Jordanian Population: A Cross-Sectional Study
Source: F1000Res. 2022 Jan 12;10:1242. Originally published 2021 Dec 6. [Version 2] doi: 10.12688/f1000research.74492.2 (PMC8672222; doi:10.12688/f1000research.74492.2)
Supplement: Peer review history

# Peer review: Reviewer response for version 2

**DOI:** 10.5256/f1000research.118400.r119568

**Published:** 2022-01-13

**Authors:** Nadeen Anabtawi

**Affiliations:** 1 Department of Pharmacology and Toxicology, Wright State University Boonshoft School of Medicine, Dayton, OH, USA

Previous comments were properly addressed. I have no further comments, and I see the revised version is fit for publication.

Is the work clearly and accurately presented and does it cite the current literature?

Yes

If applicable, is the statistical analysis and its interpretation appropriate?

Yes

Are all the source data underlying the results available to ensure full reproducibility?

Yes

Is the study design appropriate and is the work technically sound?

Yes

Are the conclusions drawn adequately supported by the results?

Yes

Are sufficient details of methods and analysis provided to allow replication by others?

Yes

Reviewer Expertise:

Pharmaceutical Science

I confirm that I have read this submission and believe that I have an appropriate level of expertise to confirm that it is of an acceptable scientific standard.

# Peer review: Reviewer response for version 2

**DOI:** 10.5256/f1000research.118400.r119570

**Published:** 2022-01-13

**Authors:** Abdelrahim Alqudah

**Affiliations:** 1 Department of clinical pharmacy and pharmacy practice, Faculty of pharmaceutical sciences, The Hashemite University, Zarqa, Jordan

I have no further comments to make.

Is the work clearly and accurately presented and does it cite the current literature?

Yes

If applicable, is the statistical analysis and its interpretation appropriate?

Yes

Are all the source data underlying the results available to ensure full reproducibility?

Yes

Is the study design appropriate and is the work technically sound?

Yes

Are the conclusions drawn adequately supported by the results?

Yes

Are sufficient details of methods and analysis provided to allow replication by others?

Yes

Reviewer Expertise:

Diabetes and cardiovascular disease

I confirm that I have read this submission and believe that I have an appropriate level of expertise to confirm that it is of an acceptable scientific standard.

# Peer review: Reviewer response for version 2

**DOI:** 10.5256/f1000research.118400.r119569

**Published:** 2022-01-13

**Authors:** Taher Hatahet

**Affiliations:** 1 School of Pharmacy, Queen's University Belfast, Belfast, UK

Approved

Is the work clearly and accurately presented and does it cite the current literature?

Yes

If applicable, is the statistical analysis and its interpretation appropriate?

Yes

Are all the source data underlying the results available to ensure full reproducibility?

Yes

Is the study design appropriate and is the work technically sound?

Yes

Are the conclusions drawn adequately supported by the results?

Partly

Are sufficient details of methods and analysis provided to allow replication by others?

Partly

Reviewer Expertise:

pharmacy education, community pharmacy, drug delivery  and nanotechnology

I confirm that I have read this submission and believe that I have an appropriate level of expertise to confirm that it is of an acceptable scientific standard.

# Peer review: Reviewer response for version 1

**DOI:** 10.5256/f1000research.78255.r102097

**Published:** 2021-12-15

**Authors:** Abdelrahim Alqudah

**Affiliations:** 1 Department of clinical pharmacy and pharmacy practice, Faculty of pharmaceutical sciences, The Hashemite University, Zarqa, Jordan

This paper discusses factors associated with knowledge and awareness of stroke among the Jordanian population which is an important topic that gives new insight about this serious complication.

 I would like to thank the authors for the great flow of their introduction. They started their introduction by giving information about stroke and its prevalence worldwide, then they focused on the effect of good knowledge and awareness of the population on the prevention of cerebrovascular events resulting from stroke. After that, they raised the issue of lack of knowledge and awareness among the population and how this can increase the risk of this serious event which supports the aim of their research.

 The study design was very good. The snowball method is a good technique to increase the response rate. The authors followed the ethical considerations in their data collection procedure. The response rate (573) is considered very good and above the required number. The used instrument was very good and well-prepared to cover the sociodemographic and knowledge levels. Their statistical model was good which used frequencies, descriptive stats and logistic regression to predict the factors affecting population knowledge about stroke. The figures and tables represented the results in a great way which makes the results clear and easy to understand. The discussion was well-structured and concise and explained all the related points mentioned in the results section.

 This study concluded that the knowledge was affected by education level, living in urban areas, and being employed, therefore, targeting a population with inadequate knowledge about stroke could reduce the risk of stroke and its complications.

 I think the results of this study will give new insights for the government to focus more on the awareness campaigns about stroke. So, I would recommend the publication of this study.

Is the work clearly and accurately presented and does it cite the current literature?

Yes

If applicable, is the statistical analysis and its interpretation appropriate?

Yes

Are all the source data underlying the results available to ensure full reproducibility?

Yes

Is the study design appropriate and is the work technically sound?

Yes

Are the conclusions drawn adequately supported by the results?

Yes

Are sufficient details of methods and analysis provided to allow replication by others?

Yes

Reviewer Expertise:

Diabetes and cardiovascular disease

I confirm that I have read this submission and believe that I have an appropriate level of expertise to confirm that it is of an acceptable scientific standard.

# Response

**Published:** 2021-12-20

**Authors:** Muna Barakat

**Affiliations:** Applied Science Private University University , Jordan

Dear Dr Abdlarahim 

 Thank you for your great feedback and review. It was a pleasure to get your attention to our work.

# Peer review: Reviewer response for version 1

**DOI:** 10.5256/f1000research.78255.r102102

**Published:** 2021-12-14

**Authors:** Taher Hatahet

**Affiliations:** 1 School of Pharmacy, Queen's University Belfast, Belfast, UK

**Factors Associated with Knowledge and Awareness of Stroke Among the Jordanian Population: A Cross-Sectional Study**

**Overview:**

 The article investigates the awareness associated with stroke in Jordan.

**In Key words**

 I suggest adding other key words like 'cross sectional study'.

**In the introduction**

 Paragraph 1: there 2 refs not inserted as hyperlinks

 “recent changes in the segment of countries” should be further explained what happened exactly and are these changes seen in Jordanian context?

 "noting that 80% of stroke cases are preventable if necessary precautions and actions are taken"

 It is better to write it as a separate sentence: "It is worth noting that..."

 “past medical history is also essential to **be** investigate **d”**

 Paragraph 4: the talk about gender covered females but did not cover males.

 Paragraph 5: please add the impact of the work in the scope of the region not only the country to highlight the article value to wider readers.

**Method section**

**Study design**

 It is not clear to how many people the survey was sent to in total and what the method of contact through social media was. This should be made clearer - I don’t think the survey reached to all internet users in the country!

 Sample size calculation

 I can see the relationship between being able to identify 3 out of 5 factors to sample size calculations?

**Results**

 Figure 1 resolution is low, the figure should be inserted as a high resolution figure.

**Discussion**

 I think some of the limitation mentioned at the limitation section should be also introduced to the discussion of results especially when presenting much higher awareness of stroke in the sample population in Jordan compared to other countries.

 It is advisable to discus not simply the questions of the survey but also the type of sample taken. Like when talking about identification of at least one risk factor. We can see the very high % in Jordan compared to Spain which I think is coming for the type of sample and this should be added to discussion. The same applies to recalling at least one stroke symptom.

 In paragraph 7: there should be more comparison to literature from the same geographic area to see if the same findings were reported, like in Lebanon or Saudi Arabia, do people living in rural areas score lower in stroke questionnaires than in urban ones? etc.

 I hope that the author would improve on the comments but keeping in mind that the paper is of good quality. I consider **Approved with Reservations** to be too much for my comments.

Is the work clearly and accurately presented and does it cite the current literature?

Yes

If applicable, is the statistical analysis and its interpretation appropriate?

Yes

Are all the source data underlying the results available to ensure full reproducibility?

Yes

Is the study design appropriate and is the work technically sound?

Yes

Are the conclusions drawn adequately supported by the results?

Partly

Are sufficient details of methods and analysis provided to allow replication by others?

Partly

Reviewer Expertise:

pharmacy education, community pharmacy, drug delivery  and nanotechnology

I confirm that I have read this submission and believe that I have an appropriate level of expertise to confirm that it is of an acceptable scientific standard.

# Response

**Published:** 2021-12-20

**Authors:** Muna Barakat

**Affiliations:** Applied Science Private University University , Jordan

Dear Dr Taher:

 Thank you for your great feedback and review. We appreciate your efforts and comments and we tried to address them carefully. 

**In Key words**

 I suggest adding other key words like 'cross sectional study'.

 R: Done

**In the introduction**

 Paragraph 1: there 2 refs not inserted as hyperlinks

 R: Done

 “recent changes in the segment of countries” should be further explained what happened exactly and are these changes seen in Jordanian context?

 R: This sentence has been removed

 "noting that 80% of stroke cases are preventable if necessary precautions and actions are taken"

 It is better to write it as a separate sentence: "It is worth noting that..."

 R: Done

 “past medical history is also essential to **be** investigate **d”**

 R: Done

 Paragraph 4: the talk about gender covered females but did not cover males.

 R: 

 he discussion was tailored to the influence of female gender as the results were significant for the influence of female rather than male.

 Paragraph 5: please add the impact of the work in the scope of the region not only the country to highlight the article value to wider readers.

 R: The following was added

 " . The study will provide an overall insight towards importance of raising the level of knowledge and awareness towards stroke to minimize stroke development, to prevent stroke recurrence, and ensure early patient presentation" 

**Method section**

**Study design**

 It is not clear to how many people the survey was sent to in total and what the method of contact through social media was. This should be made clearer - I don’t think the survey reached to all internet users in the country!

 R: The questionnaire link was sent through social media networks to gather the largest number of responses, however, through this means of communication we are not able to know the exact number of received invitations.

 Sample size calculation

 I can see the relationship between being able to identify 3 out of 5 factors to sample size calculations?

 R: 

 Based on a search that was performed to identify the highly related study to be able to calculate the target sample size, we identified the study conducted by (Sadighi et al., 2018a).

**Results**

 Figure 1 resolution is low, the figure should be inserted as a high resolution figure.

 R: A new Figure will be uploaded. 

**Discussion**

 I think some of the limitation mentioned at the limitation section should be also introduced to the discussion of results especially when presenting much higher awareness of stroke in the sample population in Jordan compared to other countries.

 R: The discussion section has been modified 

 " Although Karasneh et al 2020 mentioned in their study that Jordanians have an inadequate level of health literacy, most of the participants in our study expressed good knowledge regarding stroke (Karasneh et al., 2020)." 

 It is advisable to discus not simply the questions of the survey but also the type of sample taken. Like when talking about identification of at least one risk factor. We can see the very high % in Jordan compared to Spain which I think is coming for the type of sample and this should be added to discussion. The same applies to recalling at least one stroke symptom.

 R: It is mentioned in the discussion 

 " In our study, 98.1% of participants identified at least one risk factor related to stroke. In comparison, previous studies have reported 85.4% among 390 participants in Lebanon (Khalil and Lahoud, 2020), 76.2% among 822 participants in Australia (Sug Yoon et al., 2001), and 59.6% among 2884 participants in Spain (Segura et al., 2003). Conversely, other previous studies have demonstrated poor knowledge of stroke risk factors and symptoms in the general population (Jones et al., 2010, Stroebele et al., 2011a, Nicol and Thrift, 2005)." 

 In paragraph 7: there should be more comparison to literature from the same geographic area to see if the same findings were reported, like in Lebanon or Saudi Arabia, do people living in rural areas score lower in stroke questionnaires than in urban ones? etc.

 R: In the discussion section

 "Moreover, our results revealed that living in an urban area was significantly associated with better awareness of stroke risk factors and consequences; this could be attributed to better access to information resources and health services than rural ones (Joubert et al., 2008). Similar findings were revealed by Alluqmani 2021 in Saudi Arabia, as they also recommended for comprehensive investigation for stroke awareness, including large samples in rural populations (Alluqmani et al., 2021)."

# Peer review: Reviewer response for version 1

**DOI:** 10.5256/f1000research.78255.r102098

**Published:** 2021-12-13

**Authors:** Nadeen Anabtawi

**Affiliations:** 1 Department of Pharmacology and Toxicology, Wright State University Boonshoft School of Medicine, Dayton, OH, USA

A well-written manuscript that assessed the knowledge and awareness of stroke among the Jordanian population. Despite the limitations, which have already been addressed by the authors, this manuscript gets to a great conclusion that is well supported by extensively analyzed data. I have some minor comments below that won’t affect the robustness of the manuscript, but they are just opportunities for improvement.

 Comments:

1. The novelty of this research is not clearly stated. Earlier research (Madae’en et al., 2013) with the same purpose, which is referenced by the author and results were compared. However, I suggest explaining what are the main points that this research will add to the previous knowledge about stroke awareness in the Jordanian population and emphasizing the meaningful contribution of this manuscript to the field.
2. The study revealed a good knowledge and awareness level about stroke among Jordanians. However, “good” could be a vague term to use without further explanation. I suggest adding a description of what “good or satisfactory” exactly means and emphasizing the rating scale that resulted in such a conclusion. Is it compared to previous research and the percentage was improved, or there were specific criteria for rating the good and the bad knowledge?
3. I assumed that the American Heart Associated stroke definition was used to generate the assessment criteria used for stroke knowledge. However, it would be more reasonable to clearly state the rationale behind choosing the assessment criteria used throughout the manuscript.

Overall, this is an interesting study and the authors have collected and analyzed a good dataset using appropriate methodology. The paper is generally well written and structured, and I recommend it for publication.

Is the work clearly and accurately presented and does it cite the current literature?

Yes

If applicable, is the statistical analysis and its interpretation appropriate?

Yes

Are all the source data underlying the results available to ensure full reproducibility?

Yes

Is the study design appropriate and is the work technically sound?

Yes

Are the conclusions drawn adequately supported by the results?

Yes

Are sufficient details of methods and analysis provided to allow replication by others?

Yes

Reviewer Expertise:

Pharmaceutical Science

I confirm that I have read this submission and believe that I have an appropriate level of expertise to confirm that it is of an acceptable scientific standard.

# Response

**Published:** 2021-12-20

**Authors:** Muna Barakat

**Affiliations:** Applied Science Private University University , Jordan

Dear Dr. Nadeen

 Thank you for your great feedback and review. It was a pleasure to get your attention and we tried to address your fruitful comments carefully as the following:

 1- The discussion section has been amended as requested.

 "Although a similar study was previously conducted in Jordan by (Madae’en et al., 2013), which assessed the level of knowledge and awareness toward stroke among the general Jordanian population, this study did not evaluate the factors that exert an influential effect on stroke. Thus; our study provided insight into both the level of knowledge and awareness toward stroke and the factors associated with it."

 2-  The method section has been amended as required.

 "According to the previous study by (Han et al., 2019a), participants were awarded one point per correct answer to the above statements, however, it lacks a cutoff value that identifies the acceptable level of knowledge. Thus, our study summed up the total correct answers and considered a good level of knowledge above 50%."

 3. The definition that was utilized through the manuscript was in accordance with the definition set by the American heart association and it was applied among all manuscript sections.

# Response

**Published:** 2022-01-12

**Authors:** Nadeen Anabtawi

**Affiliations:** Wright State University, USA

Previous comments were properly addressed. I have no further comments and I see the final version is fit for publication.
